# Pandemic H1N1 virus transmission and shedding dynamics in index case households of a prospective Vietnamese cohort^☆^

**DOI:** 10.1016/j.jinf.2014.01.008

**Published:** 2014-06

**Authors:** Pham Quang Thai, Le Quynh Mai, Matthijs R.A. Welkers, Nguyen Le Khanh Hang, Le Thi Thanh, Vu Tien Viet Dung, Nguyen Thi Thu Yen, Tran Nhu Duong, Le Nguyen Minh Hoa, Dang Dinh Thoang, Hoang Thi Huyen Trang, Menno D. de Jong, Heiman Wertheim, Nguyen Tran Hien, Peter Horby, Annette Fox

**Affiliations:** a National Institute of Hygiene and Epidemiology, Hanoi, Viet Nam; b Department of Medical Microbiology, Academic Medical Center, University of Amsterdam, Amsterdam, The Netherlands; c Oxford University Clinical Research Unit and Wellcome Trust Major Overseas Programme, Viet Nam; d Ha Nam Centre for Preventive Medicine, Ha Nam, Viet Nam; e Center for Tropical Medicine, Nuffield Department of Clinical Medicine, University of Oxford, Oxford, UK; f Department of Microbiology and Immunology, University of Melbourne, Australia

**Keywords:** Influenza, Transmission, Pandemic, Serial interval, Household, Shedding, Symptoms, Index, Secondary, Antibody

## Abstract

**Objectives:**

Influenza household transmission studies are required to guide prevention strategies but most passively recruit index cases that seek healthcare. We investigated A(H1N1)pdm09 transmission in a household-based cohort during 2009.

**Methods:**

Health-workers visited 270 households weekly, and collected swabs from influenza-like-illness cases. If A(H1N1)pdm09 was RT-PCR-confirmed, all household members had symptoms assessed and swabs collected daily for 10–15 days. Viral RNA was quantified and sequenced and serology performed on pre-pandemic sera.

**Results:**

Index cases were detected in 20 households containing 81 people. 98.5% lacked A(H1N1)pdm09 neutralizing antibodies in pre-pandemic sera. Eleven (18.6%, 95% CI 10.7–30.4%) of 59 contacts were infected. Virus genetic diversity within households was negligible and less than between households. Index and secondary cases were distributed between mothers, daughters and sons, and had similar virus-RNA shedding and symptom dynamics. Fathers were rarely infected. Five secondary cases (45%) had no apparent symptoms and three shed virus before symptoms. Secondary infection was associated with index case wet cough (OR 1.56, 95% CI 1.22–1.99).

**Conclusions:**

In this cohort of A(H1N1)pdm09 susceptible persons, virus sequencing was capable of discriminating household from community transmission. Household transmission involved mothers and children but rarely fathers. Asymptomatic or pre-symptomatic shedding was common.

## Introduction

The infectiousness of influenza cases depends on the quantity and duration of virus shedding and the extent to which respiratory symptoms, such as cough, are required for virus to be transmitted. The amount of transmission will also depend on contact susceptibility, the frequency and nature of contact between infected and susceptible persons, and the use of infection prevention practices.^1–3^ Quantification of these parameters is needed to develop and estimate the efficacy of interventions that control transmission. In particular, the impact of interventions that rely on case finding, such as quarantine and provision of masks and antivirals to contacts, will depend on how much shedding and transmission occur in the absence of symptoms. Other factors such as the duration of shedding in relation to the duration of symptoms inform the duration of intervention required.^3^

Households are important sites of influenza transmission,^4^ and provide valuable information about virus transmission and shedding dynamics because contacts of index cases can often be observed before virus shedding and symptoms start. The A(H1N1)pdm09 pandemic enabled investigations of transmission when pre-existing immunity was considered to be relatively low. Numerous case ascertainment design studies were conducted whereby households are investigated following passive detection of cases presenting to health care centers,^5–13^ some of which required laboratory confirmation of secondary infection.^14–20^ Estimates of household secondary attack rate (SAR) or secondary infection risk (SIR) ranged from 3 to 38% for twelve studies that collected respiratory specimens.^21^ The factors with the greatest influence on SIR included whether the study was able to identify asymptomatic infection by collecting swabs and/or paired sera from all house members; whether index cases were detected via health systems or during outbreak investigation; and the proportion of index cases that were children. In all but a few studies^6,14,16^ some contacts used antiviral prophylaxis, which affects SIR.^8,10,13,15,19,22^ Few active case finding studies were conducted and these were in school populations during outbreaks^12,22,23^ and either retrospective^12,23^ or affected by school closure and prophylaxis.^22^ One household cohort study has been reported that used paired pre- and post-season serology to detect infections.^24^

The current study uses a prospective cohort of initially uninfected households with active case finding. This is considered to be the gold standard design for influenza household studies and should provide a relatively representative and unbiased description of transmission and shedding dynamics.^25^ The participants in this study had been enrolled in the cohort since December 2007 and most had blood samples collected and tested by serology just prior to the pandemic such that prior immune status and susceptibility could be confirmed.

## Participants

The research was approved by the institutional review board of the National Institute of Hygiene and Epidemiology, Viet Nam, the Oxford Tropical Research Ethics Committee, University of Oxford, UK. All participants provided written informed consent.

The investigations described here were conducted as part of an ongoing household-based influenza cohort study that has been described in detail elsewhere.^26^ In brief, households from a commune in Ha Nam Province, in northern Viet Nam were selected at random. 940 members of 270 randomly selected households were enrolled. Index cases were detected via active surveillance for influenza-like illness (ILI), defined as a fever >38 °C and cough, or sore throat. Health workers examined all persons in confirmed A(H1N1)pdm09 case households, including those without symptoms, each day for up to 15 days during the first pandemic wave (September–December 2009). Examinations included collection of nose- and throat-swabs for quantitative RT-PCR and full-genome sequencing; mouth temperature measurement, scored on a 5-tier scale (36–36.9 = 1, 37–37.9 = 2, 38–38.9 = 3, 39–39.9 = 4, ≥40 = 5); and evaluation of symptoms (sore throat, nasal congestion, runny nose, sneezing, dry cough, wet cough, headache, diarrhoea, myalgia, fever, and wheeze), which were scored on a 3-tier scale (none = 0, mild = 1, or moderate/severe = 2). A cough was defined as wet or productive if sputum or material from the bronchi was expectorated. Participants were also asked if they took the day off work because of illness or to care for another household member that was ill, and if they took oseltamivir. Blood samples were collected for serology in June 2009 and April 2010.

## Methods

### Virology and serology

Separate flocked swabs (Copan, Brescia, Italy) were used to firmly swab the entire posterior pharynx and tonsillar area and the nasal cavity at the level of the turbinates. Nasal and throat swabs were combined in 1 tube containing 3 ml of viral transport medium, and transferred to the laboratory within 24 h where they were vortexed before aliquoting and storing the media at −80 °C.

RNA was extracted from swab media and assessed by real-time reverse-transcriptase polymerase chain reaction (RT-PCR), according to WHO/USCDC protocols (CDC reference no. I-007-05, http://www.who.int/csr/resources/publications/swineflu/CDCRealtimeRTPCR_SwineH1Assay-2009_20090430.pdf). A cycle threshold value ≤40 was considered positive. Swabs from participants with confirmed infection were further assessed in a quantitative RT-PCR assay targeted at the M gene as described previously.^27^ The target sequence was cloned and quantified using pico green to prepare a standard curve for quantitation. Standard curves were run in duplicate. Samples were generally tested once but RT-PCR was repeated to validate fluctuations. Results were expressed as cDNA equivalent copies of viral RNA. The limit of detection was 5 RNA copies/reaction. De novo whole genome sequencing was performed on combined nose and throat swabs with Ct values below 33. All 8 virus gene segments were amplified in two RT-PCR reactions by using primers that target the conserved termini: (5′-GCCGGAGCTCTGCAGATATCAGCRAAAGCAGG-3′) or (5′-GCCGGAGCTCTGCAGATATCAGCGAAAGCAGG-3′) with (5′-CAGGAAACAGCTATGACAGTAGAAACAAGG-3′).^28^ 454 sequencing adaptors and molecular identifier tags were ligated to combined PCR products using the SPRIworks Fragment Library System II for Roche GS FLX* DNA Sequencer. Emulsion PCR, bead recovery and enrichment were performed manually according to the manufacturer's protocol followed by sequencing on a Roche GS FLX+. Analysis was limited to the envelope gene sequences in the current study. Sequences will be made available in Genbank.

Sera were tested in haemagglutination inhibition (HI) and microneutralization (MN) assay as previously described.^26^ A reference antigen supplied by WHO (A/California/7/2009(H1N1)-like) was used with turkey erythrocytes. Titres were read as the reciprocal of the highest serum dilution causing complete inhibition of agglutination. If there was no inhibition of HI at the highest serum concentration (1:10 dilution) the titre was designated as 5.

### Definitions and analysis

Influenza infection was defined as a positive RT-PCR, regardless of the presence of symptoms. Household members with RT-PCR confirmed infection but no increase in mouth temperature and none of the symptoms listed earlier were defined as asymptomatic infection. Serology was not routinely performed on acute sera so was not considered in the definition of secondary infection. Nevertheless, seroconversion was reported if there was a 4-fold or greater rise in HI or MN titre between pre- and post-pandemic sera. Household secondary infection risk (SIR) was calculated as the number of household contacts infected 1–8 days after symptom onset in the index case divided by the number of household contacts, similar to other studies.^6,7,13,15,17^ Serial interval was defined as the number of days between symptom onset in the index case and the first secondary case. Other secondary household cases were only included in the serial interval calculation if their symptoms started on the same day as the first secondary case. Children were defined as those up to 15 years of age. Oseltamivir treatment was considered to be timely if commenced within 2 days of symptom onset. Shedding time was defined as the day since onset that viral RNA could still be detected for virologically-confirmed cases that provided samples on sufficient days for negative swabs to be detected. Kaplan–Meier estimates for median time until viral RNA was undetectable (<5 copies per reaction) were determined using right censoring at the last positive sample day, and compared for cases who took timely Oseltamivir versus late or no Oseltamivir by Log Rank (Mantel–Cox) test.

Continuous variables are presented as median and interquartile ranges and compared using Rank sum test. Undetectable viral RNA levels were assigned a value of one to facilitate Log 10 transformation. Chi-squared or Fisher's exact test were used for proportions. All statistical tests were 2 sided, and probability less than 0.05 was considered significant. Univariate and multivariate logistic regression was performed to determine factors associated with A(H1N1)pdm09 infection among contacts. Generalized estimating equations were used to account for household clustering in the logistic regression model. Predictor variables included the age and sex of the contact and of the index case, number of people in the household and index case peak viral load, sum of daily scores for symptoms and antiviral treatment. Variables with a univariate *P* value <0.10 were included in multivariate analysis. The Box–Tidwell test was used to assess the assumption of linearity.^5,6^

## Results

### Index case household characteristics

Index cases were detected in 20 (7.4%) of 270 households (Table 1). Two households had two separate index case episodes resulting in 22 index cases. The second episode was excluded from analysis of transmission. The households contained 81 people including the 22 index cases with the remaining 59 classified as contacts. Households comprising four people were significantly more common than amongst all 270 cohort households (*p* = 0.009). Accordingly, most households comprised nuclear families with similar numbers of mothers, sons and daughters whereas some households lacked fathers. 25% of sons and daughters were older than 15 years. The median age of people in index case households was 23.3 years (IQR 12.2–39.3) with significantly fewer in the youngest and oldest age categories compared to all 270 households in the cohort. Pre-pandemic blood was collected from 69 (85%) of the index case household members (Table S1). HI titres against A(H1N1)pdm09-like virus were <10 in all but one who had a titre of 20 and was not infected. None reported ever having received influenza vaccine.

### Secondary cases

Eleven of 59 contacts were infected, giving a household secondary infection risk (SIR) of 18.6% (95%CI 10.7–30.4%). The secondary cases were from eight (40%) of the index case households. Five households had one secondary case, three households had two and twelve households had none. Six of the secondary cases were symptomatic giving a household secondary confirmed influenza illness risk of 10.2% (95%CI 4.8–20.5%). Five were asymptomatic, representing 45% of secondary infections. Four asymptomatically infected contacts also had blood collected for serology, of which three seroconverted (Table S1). The asymptomatic case that did not convert was an adult who had a 2-fold rise in titre, and viral RNA detected in swabs on 5 consecutive days. Her two children had virologically confirmed infection and both seroconverted but one was also asymptomatic. Six additional seroconverters were detected among 48 household members whose swabs remained negative during the period of the household transmission study. None of these six seroconverters reported ILI. In total, 69 people from index case households were assessed by serology as well as RT-PCR on swabs. Of these, 39 (56%) had virologically confirmed infection and/or seroconversion during the first pandemic wave (Table S1). Viral sequencing demonstrated that the genetic distance between haemagglutinin and neuraminidase genes of viruses from the same household was around 3–4 times less than between viruses from different households (Table 2). Analysis of virus genes indicated that 10 of 11 secondary cases were infected within the household giving an adjusted household SIR of 17.2% (95%CI 9.6–28.9%). One infected household contact, who was the index case's husband, was suspected to have acquired infection in the community because the genetic distance between his virus and the index case's virus (0.002969) was similar to that found between households. Virus from his swabs was more closely related to viruses from another household in the same village.

Demographic data for index and secondary cases are compared in Table 3. Fourteen (64%) of 22 index cases were females and a higher proportion of females than males were index cases. Only one index case was a father whereas around one third each were mothers, daughter or sons. A high proportion of child daughters were index cases (54.5%). Secondary cases comprised fairly even numbers of males and females, and the proportion of male and female contacts with secondary infections was very similar. Similar to index cases, none of the fathers was a secondary case, and the proportion of fathers that was a case was significantly lower than for mothers, daughters and sons. Roughly half of both index and secondary cases were adults although the proportion of children that were cases was high compared to adults. The median age of index (14.9 years, IQR 9.7–36.7) and secondary cases (16.9 years, IQR 9.6–34.6) was lower than for non-infected household members (34.7 years, IQR 13.8–42.5).

### Viral RNA shedding and symptom dynamics

**Figure S1:**
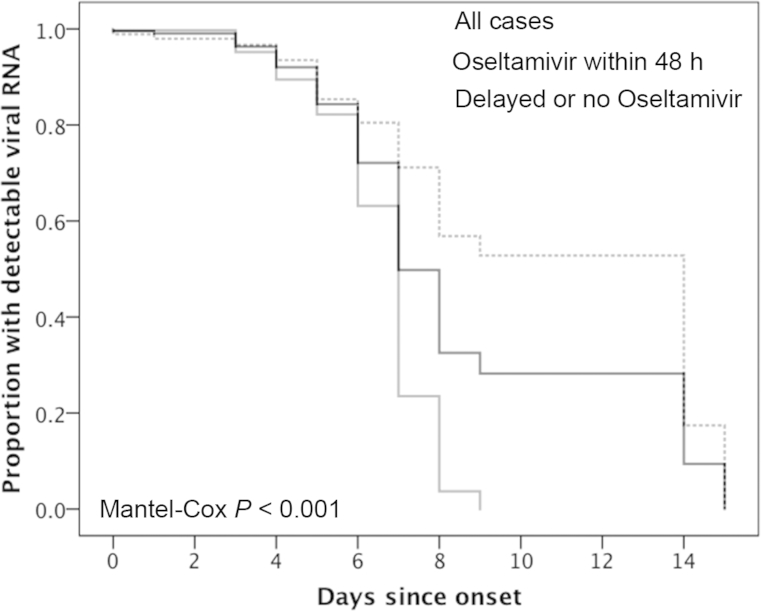
Kaplan–Meier curves of time until cessation of viral RNA shedding in virologically confirmed cases. A *P* value is shown for the comparison of cases who took timely Oseltamivir (*n* = 17) versus those who didn't take Oseltamivir or who took Oseltamivir late (*n* = 16).

**Figure 1:**
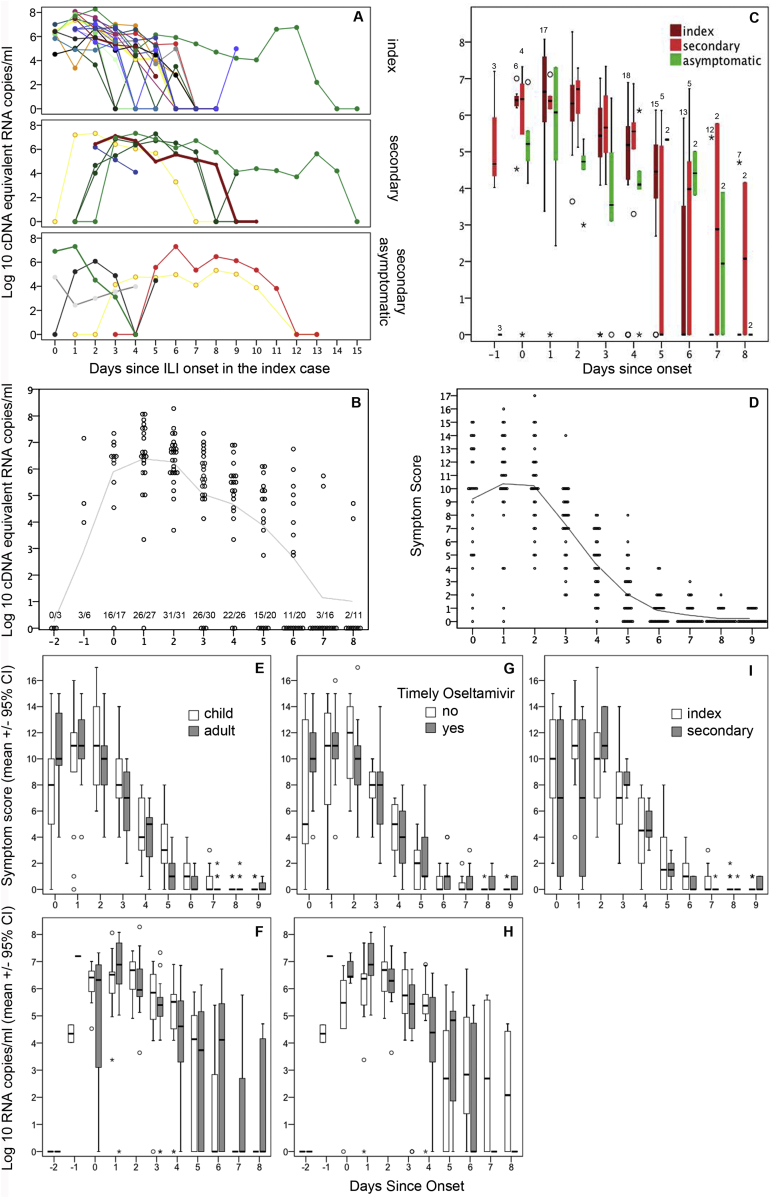
Daily viral loads and symptoms in confirmed A(H1N1)pdm09 cases from index case households. Panel A shows viral RNA shedding for each individual from index case households with virologically-confirmed infection. Participants from the same household are shown in the same colour and data is shown by day since onset in the index case to indicate the intervals between infections. Panel B shows viral RNA levels by day since onset to demonstrate viral RNA shedding dynamics. Each dot is an individual sample and the line shows the median. Fractions above the *x*-axis represent the number with detectable viral RNA over the number assessed. Panel C represents daily viral RNA levels for index cases (dark red, *n* = 20), symptomatic secondary cases (red, *n* = 6) and asymptomatic secondary cases (green, *n* = 5). Data is presented as box and whisker plots showing median lines, interquartile ranges (boxes) and ranges (whiskers). All participants in each group were tested except where numbers are shown above each bar. Panels D–I show either viral RNA shedding levels or symptom scores by day of illness for the 28 symptomatic participants. Panel D demonstrates symptom dynamics with dots representing values for individual participants and the line showing the median. Panels E and F compare adults and children. Panels G and H compare participants that took Oseltamivir within 48 h of onset versus those who took it later or did not take it. Panel I compares symptoms in index and secondary cases.

The median serial interval for symptomatic secondary cases was 2 days and ranged from 1 to 3 days (Fig. 1A, Table 4). In households with only asymptomatic secondary cases, viral RNA shedding was detected 1–5 days after symptom onset in the index case (Table 4, Fig. 1A). In 8 secondary cases the first day of viral shedding could be determined absolutely because swabs from preceding days were negative (Fig. 1A), and in three of the six with symptoms shedding commenced the day before symptoms (Fig. 1B). The vast majority of cases tested on day 0 through 2 after onset shed viral RNA (Fig. 1B). Thereafter the proportion that shed virus RNA, and levels shed, declined. The Kaplan–Meier estimate for median time until viral RNA was undetectable was 7 days (IQR 6–14 days, Fig. S1), and amongst 27 cases in whom the last shedding day could be observed the median viral RNA shedding time was 6 days with no clear difference in shedding times between symptomatic and asymptomatic cases (Table 4, Fig. 1A & C). However, both peak and day 2 viral loads were higher in symptomatic compared to asymptomatic cases. In most symptomatic cases viral RNA shedding peaked at around the time that symptoms scores peaked on day 1 and 2 after onset (Fig. 1B, C & D). Amongst cases that had symptoms there were no clear differences in virus shedding or symptom score between adults and children (Fig. 1E & F), or between index and secondary cases (Fig. 1C & I). However, three secondary cases had only a modest elevation of mouth temperature while the other three had mouth temperatures above 38 °C and classic ILI. None of the symptomatic cases required hospitalization.

Vietnamese government policy during the first wave of the A(H1N1)pdm09 pandemic dictated that all symptomatic cases should be given oral oseltamivir for 5 days. Accordingly 20 cases took oseltamivir for 5 days after symptoms developed, of whom 17 commenced by day 2 after onset (timely) and three commenced 4 days after onset. Participants with asymptomatic infection did not take oseltamivir. Cases that had timely treatment tended to have more severe symptoms and higher viral loads until the day after onset but not thereafter (Fig. 1G & H). Kaplan–Meier estimates for time until viral RNA shedding ceased were 7 days (IQR 6–7 days) for patients who took timely Oseltamivir and 14 days (IQR 7–14 days) in those who took Oseltamivir late or did not take Oseltamivir (*P* < 0.001, Fig. S1). Shedding persisted until day 13 after symptom onset in two cases from one household (Fig. 1A). Both commenced oseltamivir late. These two cases also had the highest wheeze scores, oral temperature was above 38 °C for 5 days, and daily symptom scores were relatively high. Viral sequencing did not reveal any mutations known to be associated with virulence.

### Risk factors for secondary infection

Secondary infection of household contacts was associated with index case wet cough score and viral load in univariate analysis, although paradoxically the association with viral load was negative (Table S2). Other index case symptoms and index case and contact characteristics were not significant in univariate analysis (Table S2), however numbers are small. Although contact age and number of people in the household were not significant in univariate analysis, they were included in multivariate analysis because several other studies demonstrated an association.^8,13,17^ In multivariate analysis (Table 5) infection of contacts was positively associated with the index case wet cough score (OR 1.56, 95% CI 1.22–1.99) and negatively associated with number of people in the household (OR 0.20, 95% CI 0.08–0.48). The effect of contact age was small and not significant. The association between index case viral load and contact infection was not maintained in multivariate analysis.

## Discussion

The current study sought to systematically detect A(H1N1)pdm09 index cases within a random household cohort and then intensively investigate viral RNA shedding and symptoms in household members to obtain unbiased estimates of transmission. The vast majority of household members appeared to be susceptible to infection based on pre-pandemic A(H1N1)pdm09 HI and MN titres. Eleven household contacts were infected, but 5 (45%) did not develop symptoms. Virus genetic sequencing indicated that 10 (91%) were infected within the household rather than from the community, enabling a more precise estimate of SIR. The majority of transmission involved mothers and children with a serial interval of around 2 days. The study was not powered to identify small effects on transmission but wet cough in the index case was found to have a significant effect. Studies such as this are also essential to provide precise estimations of incubation period, duration of virus shedding and relation of shedding to symptoms.

In the current study index and secondary cases were similar in terms of age, virus RNA shedding and symptoms. In contrast, studies using case ascertainment designs report a tendency for more severe symptoms and higher viral shedding for index cases,^15,16^ a bias that could lead to over-inflated SIR estimates. Factors other than severity can also influence health care seeking, leading to bias in case ascertainment studies. Surveys conducted in France and England during the A(H1N1)pdm09 pandemic found that the proportion of self-defined ILI cases that sought care was highest for children and males aged below 25 years.^29,30^

The cohort study design used here facilitated confirmation of susceptibility to infection by serology on pre-pandemic sera. Nevertheless, some index case household members may have had asymptomatic or mild infection before the index case was detected because they seroconverted without ILI or detection of virologically confirmed infection during investigation of the index case episode. This scenario would mean that fewer were susceptible. Virus genetic sequencing enabled discrimination of household from community transmission and we demonstrated that one index case household member was infected in the community rather than in the household. The within and between household genetic diversity is in agreement with other studies,^31–34^ and the magnitude of sequence diversity within individuals, households and between households was consistent with the study of Poon et al.^33^ Pascalis et al. found evidence of changes in quasi-species dominance within individuals,^34^ and we will perform further analysis of deep sequences to describe quasi-species in future. The results demonstrate that intensive investigations involving serology, virology and phylogenetics are required to obtain an accurate estimate of transmission.

A notable feature of the current study was the predominance of females amongst index cases, whereas most other A(H1N1)pdm09 transmission studies found that roughly half of index cases were females. In relation, the number and proportion of fathers infected was significantly lower than for mothers and children. Similarly, a study that assessed household contacts of children identified by active case finding during a school camp outbreak found significantly lower infection amongst fathers.^8^ These findings are also reminiscent of cohort and other studies from the 1950s^35–37^ suggesting that the pattern of transmission between mothers and children, with sparing of fathers may be a common phenomenon. Fathers in our study did not appear to be less susceptible on the basis of serology implying that they may have less exposure to infection, either via less contact with cases and/or more effective prevention of infection upon exposure. During a survey in 2007, 43% of fathers in the cohort said they cared for children compared to 55% for mothers. This difference is unlikely to account for the difference in proportion infected, but may not reflect care patterns for sick children. During the school camp outbreak study described above, 66% of the household contacts that cared for index cases were mothers, 24% were fathers and 3% were siblings.^8^

A high proportion of child daughters were index cases. It is generally considered that children are the main influenza transmitters because they have more contacts outside the house, are more susceptible to infection and severity, and shed more virus.^38^ We did not detect significant differences in virus RNA shedding or symptom scores between children and adults, similar to other studies.^20,39^ A systematic review also concluded that shedding duration of influenza A(H1N1)pdm09 was no longer among children compared with adults, either between or within studies.^40^ Perhaps susceptibility to novel virus is more uniform in accordance with the uniform absence of HI antibodies. It should also be noted that viral RNA shedding may not reveal differences in shedding of viable virus, which is relatively shorter in duration.^20^ Contact patterns could influence who is infected as an index or household secondary case. A previous study of contact patterns for this cohort demonstrated that children have the highest numbers of close contacts, both with peers and parents,^2^ but did not differentiate by gender or position in the family. Further verification of contact patterns for different family members, particularly mothers versus fathers, is planned.

Virus RNA shedding dynamics correlated with symptom scores and were generally consistent with reports elsewhere.^14–16,20^ The duration of viral RNA shedding was within the 3–9 day range reported by other studies of cases in the community.^40^ The serial interval was slightly shorter than in other studies but was based on a small number of secondary cases while tertiary cases were excluded. As noted by Lau et al., serial interval estimates could be shortened by correction for multiple chains of transmission (e.g., tertiary cases), and serial interval estimates are not constant because they reflect a combination of the profile of index cases, contact patterns within households, and incubation period.^21^

Timely oseltamivir treatment of index cases was not significantly associated with infection of contacts, as reported elsewhere.^13^ However, cases that took oseltamivir early tended to have higher viral RNA shedding and symptom scores at onset compared to untreated or late-treated cases, whereas levels were similar or lower by day 2. Therefore, timely treatment may have helped to resolve shedding and symptoms.

Forty five percent of virologically confirmed household secondary cases did not develop symptoms, higher than reported by others.^6,14,18,20,39^ One asymptomatic case did not seroconvert, which may indicate that viral RNA remained in the respiratory tract without being internalized and eliciting an immune response. Contrary to expectations, the duration of viral RNA shedding was similar for symptomatic cases and asymptomatic cases, perhaps because asymptomatic cases did not take oseltamivir. In contrast Loeb et al. reported a shorter duration of shedding in asymptomatic cases.^39^

The extent to which shedding without symptoms contributes to influenza transmission is unclear.^41^ A few studies have investigated transmission during pre-symptomatic shedding in humans, but involve only a few index cases, rely on recall, and can't control for exposure.^42,43^ One study has demonstrated transmission before symptoms in ferrets.^44^ Virus emission is an important component of transmission and is related to both nasopharangeal viral load and the mechanical processes of coughing and sneezing.^45^ In the current study viral RNA shedding was lower in asymptomatic compared tosymptomatic cases, consistent with Loeb et al.,^39^ but in contrast to Suess et al.^20^ Household transmission was also associated with the amount of wet cough in the index case, consistent with several other studies,^11,13,17^ and suggesting that transmission from symptomatic cases is more efficient. However, virus emission has been reported to vary substantially between individuals,^45^ and this could confound our interpretation of risk factors. Further definition of the contribution of shedding without or before symptoms to transmission is required to estimate the effectiveness of control measures such as case quarantine and timely treatment.

The major limitations of the current study were the small number of index cases, and the selection of households from just one commune. Although nearly 1000 people were included in the cohort, the number of index cases could not be controlled and was not sufficient to assess risk factors for transmission, particularly factors with a lot of variance such as viral load. Households were selected from one commune because we lacked sufficient resources to maintain intensive surveillance in multiple sites, representative of the population. Nevertheless, the commune was representative of a large proportion of the population that reside within the semi-rural deltas. Studies are underway to investigate urban versus rural differences in transmission and contact patterns.

This cohort study avoided many of the limitations of other studies of A(H1N1)pdm09 transmission in households including case ascertainment bias, assumptions about immunity/susceptibility and transmission within the household, and failure to detect asymptomatic infection.^21,25^ Cohort studies are resource and labour intensive but can provide more reliable estimates of SIR. The intensive assessment of shedding and symptoms demonstrated that a substantial amount of shedding occurs without symptoms but wet cough in the index case was associated with significantly increased transmission.

## Figures and Tables

**Table 1:** Composition of households in the cohort and those with an index case.

		All houses *n* (%)	Index houses *n* (%)	*p* value
Houses		270	20	
People		940	81	

People per house	1	28 (10.4)	0 (0)	–
	2	41 (15.2)	1 (5)	0.327
	3	65 (24.1)	4 (20)	0.792
	4	74 (27.4)	11 (55)	0.009
	5	42 (15.6)	3 (15)	1.000
	≥6	20 (7.4)	1 (5)	1.000

Females		508 (54.5)	42 (51.9)	0.704

Position in the household/family	Mother	250 (26.6)	20 (24.7)	0.756
	Father	207 (22.0)	15 (18.5)	0.496
	Daughter	204 (21.7)	20 (24.7)	0.494
	Son	183 (19.5)	22 (27.2)	0.085
	Other	83 (8.8)	3 (3.7)	0.116
	Unknown	14 (1.5)	1 (1.2)	1.000

Age	0–4	83 (8.9)	2 (2.5)	0.049
	5–9	70 (7.5)	10 (12.3)	0.107
	10–19	209 (22.5)	25 (30.9)	0.066
	20–39	246 (26.5)	25 (30.9)	0.323
	40–59	241 (25.9)	17 (21.0)	0.386
	≥60	80 (8.6)	1 (1.2)	0.021
	Unknown		1 (1.2)	

**Table 2:** Comparison of H1N1-2009 envelope gene sequence diversity within households and individuals and between households.

	Mean *p*-distance^a^ (standard deviation)	
	Haemagglutinin	Neuraminidase
Within an individual	0.00007215 (0.000161)	0.00004304 (0.000143)
Within a household^b^	0.000509 (0.001107)	0.000608 (0.001322)
Between households^b^	0.002262 (0.001140)	0.002280 (0.000908)

a *p*-distance is the number of nucleotide substitutions divided by the number of nucleotides calculated using Mega version 5.2. *p*-distance values were similar to *d*-distance values, which correct for ‘unmeasured’ nucleotide changes using the nucleotide substitution Kimura-2-parameter model.

b Only the first time point of each infected participant was used.

**Table 3:** Distribution of cases, contacts and secondary cases by age, gender and position in the family.

	All house members			Contacts
	*N*	Any case^a^	Index case^a^	Secondary case
		*n* (%)	*n* (%)	*n*/*N* (%)
Child	30	16 (53.3)	11 (36.7)	5/19 (26.3)
Adult	50	17 (34.0)	11 (22.0)	6/39 (15.4)^b^

Female	42	19 (45.2)	14 (33.3)	5/28 (17.9)
Male	39	14 (35.9)	8 (20.5)	6/31 (19.3)^b^

Mother	20	9 (45.0)	6 (30.0)	3/14 (21.4)
Father	15	1 (6.7)^c^	1 (6.7)	0/14 (0)
Child daughter	11	7 (63.6)	6 (54.5)	1/5 (20.0)
Adult daughter	9	3 (33.3)	2 (22.2)	1/7 (14.3)
Child son	18	9 (50.0)	5 (27.8)	4/13 (30.8)
Adult son	4	3 (75.0)	2 (50.0)	1/2 (50.0)
Other	3	1 (33.3)	0 (0.0)	1/3 (33.3)^b^

a The denominator is the number of household members in each category; demographic data was incomplete for 1 household member.

b HA and NA gene sequences indicate that one case may have been infected in the community, who was an adult male whose position in the family is other.

c The proportion of fathers with virologically-confirmed infection was significantly lower (*Χ*^2^*p* = 0.021) compared to mothers (OR 11.45, 95% CI 1.25–104.60), daughters (OR 14.00. 95% CI 1.54–127.62) and sons (OR 16.80, 95% CI 1.87–150.94).

**Table 4:** Virus shedding and transmission characteristics.

	Index (*n* = 18)	Secondary (*n* = 6)	Asymptomatic (*n* = 5)
Serial Interval	NA	1, 1, 2, 2, 3, 3	1, 1, 1, 5
Shedding Days^a^	6.0 (4.0–7.0)	6.5 (6.0–8.8)	6.0 (4.0–7.0)
Peak Log 10 Viral Load	7.0 (6.6–7.4)	7.2 (6.6–7.6)	6.1 (5.0–7.3)
Day 2 Log 10 Viral Load	5.6 (4.6–6.4)	6.4 (4.8–6.6)	4.7 (3.3–5.1)^*p* = 0.038^

Results are presented as median and interquartile range in brackets or as values for individuals.

a 4 index cases, 1 secondary case and 1 asymptomatic case were excluded because insufficient samples were collected to assess shedding time.

**Table 5:** Risk factors for transmission of H1N1-2009 from index case to household contacts during the first pandemic wave.

Variable	Contact status^a^		OR (CI)	*p*	Adjusted OR (CI)	*p*
	Infected (*n* = 11)	Not infected (*n* = 47)				
Contact age	16.9 (9.6–34.6)	31.9 (13.9–41.9)	0.96 (0.92–1.01)	0.112	0.94 (0.88–1.01)	0.115
Index Peak Log 10 Viral load^b^	6.4 (5.8–7.3)	7.0 (6.7–7.5)	0.33 (0.12–0.86)	0.020	0.56 (0.14–2.23)	0.409
Index wet cough^c^	8 (3–10)	4 (0–7)	1.36 (1.07–1.72)	0.012	1.56 (1.22–1.99)	<0.001
People/house	4 (3–4)	4 (4–5)	0.46 (0.17–1.29)	0.140	0.20 (0.08–0.48)	<0.001

a Results are presented as median and interquartile range.

b Maximum Log 10 cDNA equivalent viral RNA copies/ml detected for each index case.

c Summed score for wet cough over the course of illness in the index case ranging from 0 for no cough to 2 for moderate to severe cough.
