# Supplementary material for: Pandemic H1N1 virus transmission and shedding dynamics in index case households of a prospective Vietnamese cohort
Source: J Infect. 2014 Jun;68(6):581–90. doi: 10.1016/j.jinf.2014.01.008 (PMC4031397; doi:10.1016/j.jinf.2014.01.008)
Supplement: Supplementary file 1 [file mmc1.docx]

**Supplementary Table 1.** HI and MN antibody titers in pre-pandemic, acute infection and post-pandemic sera for 81 people from index case households

|  | **HI titer** ^a^ |  | **MN titer** ^a^ |  |  |
| --- | --- | --- | --- | --- | --- |
| **Participant Infection Status** | **pre-pandemic** | **post-pandemic** | **pre-pandemic** | **post-pandemic** | **convert** |
| index | 5 | 160 | 5 | 960 | yes |
| index | 5 | 160 | 5 | 320 | yes |
| index | 5 | 320 | 5 | 640 | yes |
| index | 5 | 80 | 5 | 320 | yes |
| index | 5 | 80 | 5 | 120 | yes |
| index | 5 | 40 | 5 | 160 | yes |
| index | 5 | 40 | 5 | 80 | yes |
| index | 5 | 40 | 5 | 40 | yes |
| index | 5 | 40 | 5 | 80 | yes |
| index | 5 | 40 | 5 | 160 | yes |
| index | 5 | 40 | 5 | 160 | yes |
| index | 5 | 20 | 5 | 80 | yes |
| index | 5 | *5* | 5 | 320 | yes |
| index | 5 | *5* | 5 | 40 | yes |
| index | 5 | *5* | 5 | 320 | yes |
| index | 5 | *5* | 5 | 40 | yes |
| index | - | - | - | - |  |
| index | - | - | - | - |  |
| index | - | - | - | - |  |
| index | - | - | - | - |  |
| index | 5 | - | 5 | - |  |
| index | 5 | - | 5 | - |  |
| secondary | 5 | 40 | 5 | 240 | yes |
| secondary | 5 | 320 | 5 | 640 | yes |
| secondary | 5 | 160 | 5 | 640 | yes |
| secondary | 5 | 40 | 5 | 160 | yes |
| secondary | 5 | - | 5 | - |  |
| secondary | 5 | - | 5 | - |  |
| secondary asymptomatic | 5 | 10 | 5 | 10 | no |
| secondary asymptomatic | 5 | 320 | 5 | 640 | yes |
| secondary asymptomatic | 5 | 100 | 15 | 480 | yes |
| secondary asymptomatic | 5 | 10 | 5 | 60 | yes |
| secondary asymptomatic | 5 | - | 5 | - |  |
| RT-PCR negative | 5 | 80 | 5 | 320 | yes |
| RT-PCR negative |  | 40 |  | 120 | yes |
| RT-PCR negative | 5 | 20 | 5 | 80 | yes |
| RT-PCR negative | 5 | 5 | 5 | 80 | yes |
| RT-PCR negative | 5 | 5 | 5 | 160 | yes |
| RT-PCR negative | 5 | 5 | 5 | 120 | yes |
| RT-PCR negative | 20 | 20 | 40 | 40 | no |
| RT-PCR negative | 5 | 5 | 5 | 5 | no |
| RT-PCR negative | 5 | 5 |  | - | no |
| RT-PCR negative | 5 | 5 | 10 | 5 | no |
| RT-PCR negative | 5 | 5 | 15 | 20 | no |
| RT-PCR negative | 5 | 5 | 5 | 5 | no |
| RT-PCR negative | 5 | 5 | - | 5 | no |
| RT-PCR negative | 5 | 5 | 5 | 5 | no |
| RT-PCR negative | 5 | 5 | 5 | 5 | no |
| RT-PCR negative | 5 | 5 | 5 | 5 | no |
| RT-PCR negative | 5 | 5 | - | 5 | no |
| RT-PCR negative | 5 | 5 | 5 | 5 | no |
| RT-PCR negative | 5 | 5 | 5 | 5 | no |
| RT-PCR negative | 5 | 5 | 5 | 5 | no |
| RT-PCR negative | 5 | 5 | 15 | 10 | no |
| RT-PCR negative | 5 | 5 | - | 5 | no |
| RT-PCR negative | 5 | 5 | - | - | no |
| RT-PCR negative | 5 | 5 | - | - | no |
| RT-PCR negative | 5 | 5 | - | - | no |
| RT-PCR negative | 5 | 5 | 20 | 20 | no |
| RT-PCR negative | 5 | 5 | 5 | 5 | no |
| RT-PCR negative | 5 | 5 | 5 | 5 | no |
| RT-PCR negative | 5 | 5 | 8 | 5 | no |
| RT-PCR negative | 5 | 5 |  | - | no |
| RT-PCR negative | 5 | 5 | 5 | 5 | no |
| RT-PCR negative | 5 | 5 | 5 | 5 | no |
| RT-PCR negative | 5 | 5 | 5 | 5 | no |
| RT-PCR negative | 5 | 5 | 5 | 5 | no |
| RT-PCR negative | 5 | 5 | 5 | 5 | no |
| RT-PCR negative | 5 | 5 | 5 | 10 | no |
| RT-PCR negative | 5 | 5 | - | 5 | no |
| RT-PCR negative | - | 40 | - | - |  |
| RT-PCR negative | -- | - | - | - |  |
| RT-PCR negative | - | - | - | - |  |
| RT-PCR negative | 5 | - | - | - |  |
| RT-PCR negative | - | - | - | - |  |
| RT-PCR negative | - | - | - | - |  |
| RT-PCR negative | - | - | - | - |  |
| RT-PCR negative | - | - | - | - |  |
| RT-PCR negative | 5 | - | - | - |  |
| RT-PCR negative | 5 | - | - | - |  |
| RT-PCR negative | 5 | - | - | - |  |

a: reciprocal titers are presented as the mean of two measurements
