# Supplementary material for: Pandemic H1N1 virus transmission and shedding dynamics in index case households of a prospective Vietnamese cohort
Source: J Infect. 2014 Jun;68(6):581–90. doi: 10.1016/j.jinf.2014.01.008 (PMC4031397; doi:10.1016/j.jinf.2014.01.008)
Supplement: Supplementary file 2 [file mmc2.docx]

**Supplementary Table 2** Univariate analysis of factors associated with transmission of H1N1-2009 from index cases to household contacts during the first pandemic wave

|  |  | **Contact status** | |  |  |
| --- | --- | --- | --- | --- | --- |
| ***Variable*** |  | **Infected (11)** | **Not infected (47)** | ***OR (CI)*** | **p** |
| Contact | age | 16.9 (9.6-34.6) | 31.9 (13.9-41.9) | 0.96 (0.92-1.01) | 0.112 |
|  | Female, n/N (%) | 5/11 (45) | 23/48 (48) | 0.91 (0.20-4.05) | 0.897 |
| Index | peak Log10 Viral load^a^ | 6.4 (5.8-7.3) | 7.0 (6.7-7.5) | 0.33 (0.12-0.86) | 0.020 |
|  | wet cough score | 8 (3 -10) | 4 (0-7) | 1.36 (1.07-1.72) | 0.012 |
|  | sneeze score | 6 (3-7) | 6 (3-9) | 0.85 (0.67-1.07) | 0.226 |
|  | running nose | 5 (3-8) | 3 (1-8) | 1.09 (0.89-1.34) | 0.443 |
|  | dry cough | 0 (0-10) | 6 (0-9) | 0.88 (0.75-1.04) | 0.265 |
|  | Oseltamivir, n/N (%) | 9/11 (82) | 36/48 (75) | 1.50 (0.14-15.75) | 0.735 |
|  | age | 13.2 (8.3-33.3) | 12.4 (8.0-22.0) | 1.01 (0.96-1.07) | 0.585 |
|  | Female, n/N (%) | 8/11 (73) | 25/48 (52) | 2.45 (0.43-13.93) | 0.311 |
| House | People/house | 4 (3-4) | 4 (4-5) | 0.46 (0.17 - 1.29) | 0.140 |
|  | Child/house | 2 (1-2) | 2 (1-3) | 0.62 (0.31-1.23) | 0.168 |
